# Chronic obstructive pulmonary disease and acute myocardial infarction: effects on presentation, management, and outcomes

**DOI:** 10.1093/ehjqcco/qcw005

**Published:** 2016-02-04

**Authors:** Kieran J. Rothnie, Jennifer K. Quint

**Affiliations:** 1 Respiratory Epidemiology, Occupational Medicine and Public Health, National Heart and Lung Institute, Imperial College London, Emmanuel Kaye Building, London SW3 6LR, UK; 2 Faculty of Epidemiology and Population Health, London School of Hygiene & Tropical Medicine, London, UK

**Keywords:** Chronic obstructive pulmonary disease, Management of acute coronary syndromes, Co-morbidity, Mortality, Epidemiology

## Abstract

Cardiovascular disease is a common cause of death in patients with chronic obstructive pulmonary disease (COPD) and is a key target for improving outcomes. However, there are concerns that patients with COPD may not have enjoyed the same mortality reductions from acute myocardial infarction (AMI) in recent decades as the general population. This has raised questions about differences in presentation, management and outcomes in COPD patients compared to non-COPD patients. The evidence points to an increased risk of death after AMI in patients with COPD, but it is unclear to what extent this is attributable to COPD itself or to modifiable factors including under-treatment with guideline-recommended interventions and drugs. We review the evidence for differences between COPD and non-COPD patients in terms of the presentation of AMI, its treatment, and outcomes both in hospital and in the longer term.

## Background

Recent decades have seen substantial reductions in the incidence of acute myocardial infarction (AMI) and its mortality.^1,2^ Much of the decrease in incidence has been attributable to a decrease in ST-elevation myocardial infarction (STEMI). Rates of non-ST elevation myocardial infarction (NSTEMI) have not decreased and may be increasing perhaps due to population ageing or clinical awareness.^3^ Patients with NSTEMI tend to be older and have more co-morbidities than patients with STEMI, increasing their risk of death in the longer term.^4^ Much of the decrease in AMI mortality has been attributed to improvements in care, but it is not clear if this has been optimized for all patient groups.^5^ Some high-risk groups have received particular attention and in diabetes, for example, ischaemic presentations may be atypical and thresholds for investigation and treatment are set at a lower level compared with non-diabetic patients.^6^ However, other groups have received less attention and chronic obstructive pulmonary disease (COPD), in particular, has been under-studied in patients with AMI despite it being common, affecting ∼1.5% of the European population, although the true prevalence may be as high as 10% as many patients remain undiagnosed.^7^ In the developed world, the most important risk factor for COPD is tobacco smoking; other risk factors include increased age, indoor and outdoor pollution, poor nutrition, and low socio-economic status.^8^

**Figure 1:**
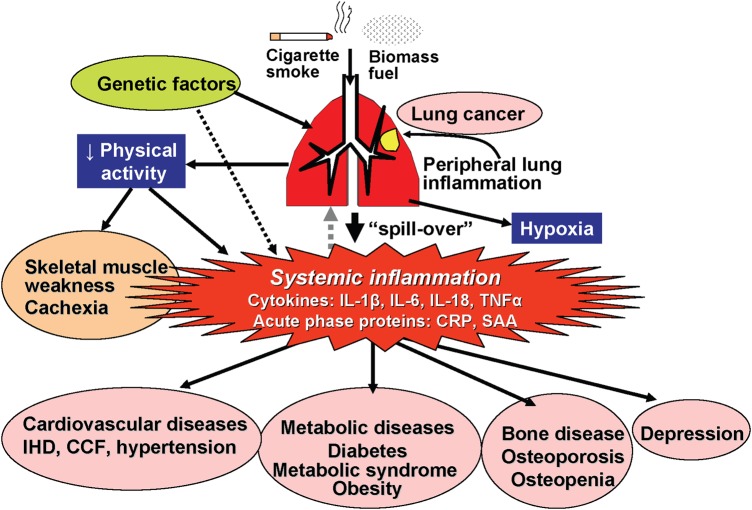
Diagram representing how inflammation in chronic obstructive pulmonary disease may ‘spill over’ into the systemic circulation and increase the risk of several diseases including cardiovascular disease. Original image from Barnes.^9^

Chronic obstructive pulmonary disease is associated with an increased risk of many other diseases, which are thought to be due, in part, to ‘spill over’ of inflammation in the lung to the systemic circulation^9^ (*Figure 1*). Cardiovascular disease is perhaps the most common co-morbidity and people with COPD, particularly in younger age groups, are at increased risk of AMI, independent of smoking status.^10,11^ Inflammation, endothelial dysfunction, and increased arterial stiffness, in addition to shared risk factors, are all thought to contribute to cardiovascular risk in COPD.^12^ Most people with COPD do not die from respiratory diseases,^13^ with cardiovascular disease being a major cause, accounting for ∼30% of all deaths.^14^

This article will review contemporary literature on how COPD affects the presentation, management, and outcomes of AMI and how these may be interrelated.

## Presentation of acute myocardial infarction

The prevalence of previously diagnosed COPD among patients presenting to hospital with AMI has been estimated as 10–17%.^15–18^ The true prevalence, including patients with undiagnosed COPD, may be significantly higher. Several studies have reported that patients with COPD are less likely to present with typical chest pain than patients without COPD, and are more likely to present with breathlessness, atypical chest pain, and palpitations.^15–17^ They are also more likely than patients without COPD to present with NSTEMI than STEMI and to have lower diagnostic biomarker levels, including troponin and creatine kinase.^18–21^ In one study, COPD has been associated with late presentation >12 h after onset of symptoms.^21^

## Recognition and management of acute myocardial infarction

One possible consequence of the differences in presentation of AMI in COPD is that its recognition is delayed or missed altogether. Around 8% of patients admitted to hospital with an acute exacerbation of COPD meet the Universal Definition for Myocardial Infarction,^22^ but it is unclear whether this represents type 2 AMI triggered by the exacerbation or type 2 AMI misdiagnosed as the exacerbation. At least 33% of patients admitted with COPD in whom there is evidence of prior AMI have no recorded cardiac diagnosis and the proportion is even higher among women with COPD.^23^ The erroneous attribution of symptoms to COPD rather than AMI may delay diagnosis and the delivery of reperfusion therapy with adverse consequences for infarct size and prognosis. In an analysis of over 300 000 first AMIs in the UK, Rothnie *et al*.^18^ found that COPD patients presenting with STEMI were more likely to have an initial incorrect diagnosis and a longer median time to reperfusion compared with patients without COPD [153 min (IQR, 74–706 min) vs. 109 min (IQR, 50–260 min)]. The difference persisted after adjustment for age, sex, and co-morbidities and was only apparent in those COPD patients in whom diagnosis was delayed.

Recent studies conducted in Sweden and the UK have shown that patients with COPD are less likely than patients without COPD to receive primary percutaneous intervention (pPCI) or other reperfusion strategies after a STEMI,^16,18^ confirming earlier US studies.^17,21^ A more recent US study, however, found no difference in rates of pPCI between patients with and without COPD, suggesting a change in practice and emphasizing the importance of observational data for identifying inequalities in patient management.^20^

NSTEMI guidelines recommend in-hospital cardiac catheterization within 72 h for patients with a ≥3% predicted risk of death at 6 months.^24,25^ Percutaneous intervention, as indicated, improves outcomes, patients at highest risk having most to gain from the intervention.^26,27^ Several studies have shown that patients with COPD who present with NSTEMI are less likely to receive in-hospital angiography compared with patients without COPD, despite being at higher risk.^16–18,20,21^ A potential explanation for this difference is that COPD patients are older and more likely to be deemed sicker or frailer than non-COPD patients, and not appropriate for more aggressive intervention. However, when comparisons are made after exclusion of patients inappropriate for angiography due, for example, to advanced cancer or dementia, findings of under-treatment are unchanged and rates of angiography remain lower in patients with than without COPD.^18^

Under-treatment of patients with COPD presenting with AMI extends beyond the acute phase. Contemporary guideline recommendations, based on randomized clinical trials, are for secondary prevention treatment with a β-blocker, an ACE inhibitor or angiotensin receptor blocker, a statin, and dual antiplatelet therapy (aspirin indefinitely and P2Y_12_ receptor antagonist for 1 year) for all patients with AMI unless there are clear contraindications.^24,25^ It has been the widely held belief that COPD contraindicates treatment with β-blockers because of the potential risk of bronchospasm caused by unopposed activation of α1 adrenergic receptors that result in smooth muscle constriction. However, many studies have shown that cardioselective β-blockers that are primarily active at cardiac β1 receptors, not bronchial β2 receptors, are not associated with a change in FEV_1_ or an increase in exacerbations of COPD.^28^ Despite this, β-blockers continue to be underused in patients with COPD who are less likely than patients without COPD to receive a prescription after AMI.^16–18,21^ The under-treatment of patients with COPD extends to other secondary prevention drugs, all of which, with the exception of P2Y_12_ receptor antagonists, tend to be prescribed less commonly in patients with COPD, although the differences are less marked compared with β-blockers.^16–21^ Findings from studies that have investigated differences in treatment between COPD and non-COPD patients after AMI are summarized in *Table 1*. Interestingly, differences in management between COPD and non-COPD patients are not apparent in all settings and appear to have changed over time. As previously mentioned, differences in rates of pPCI between patients with and without COPD appear to have narrowed over time in the USA,^20^ where prescription of β-blockers to patients with COPD has increased unlike in Europe.^16–18^ These differences between countries suggest two things: that differences in treatment between COPD and non-COPD patients do represent under-treatment, and that change is possible.

**Table 1:** Summary of studies that investigated differences in treatment after myocardial infarction between chronic obstructive pulmonary disease and non-chronic obstructive pulmonary disease patients

Study	Design and setting	Population	Differences in management
Andell *et al.* 2014^16^	Cohort study within the Swedish SWEDEHEART registry between 2005 and 2010	Consecutive patients admitted to Swedish coronary care units. COPD diagnosis ascertained through linkage to the Swedish National Patient Registry	*In-hospital management* Percutaneous coronary intervention COPD: 37.7% Non-COPD: 55.7% *P* < 0.001 Coronary angiography COPD: 72.5% Non-COPD: 55.4% *P* < 0.001 *Discharge medicines* ACE inhibitors COPD: 50.6% Non-COPD: 55.5% *P* < 0.001 Angiotensin receptor blockers COPD: 12.6% Non-COPD: 11.1% *P* = 0.001 Aspirin COPD: 85.5% Non-COPD: 90.1% *P* < 0.001 β-Blockers COPD: 77.7% Non-COPD: 86.1% *P* < 0.001 Statin COPD: 68.4% Non-COPD: 79.2% *P* < 0.001 P2Y12 inhibitor COPD: 62.5% Non-COPD: 72.2% *P* < 0.001
Bursi *et al.* 2010^21^	Cohort study in Olmsted County, MN from 1979 to 2007	3438 local residents in Olmsted County. ICD-10 codes used to ascertain COPD	*In-hospital management* Reperfusion COPD: 41% Non-COPD: 52% *P* < 0.01 Angiography in-hospital COPD: 51% Non-COPD: 59% *P* < 0.01 *Discharge medicines* ACE inhibitor COPD: 37% Non-COPD: 29% *P* < 0.01 β-Blocker COPD: 47% Non-COPD: 61% *P* < 0.01 Diuretic COPD: 34% Non-COPD: 23% *P* < 0.01 Statin COPD: 29% Non-COPD: 30% *P* = 0.61
Enriquez *et al.* 2013^20^	Cross-sectional study of National Cardiovascular Data Registry in the USA between January 2008 and December 2010	158 890 patients with an acute MI. Chronic obstructive pulmonary disease was ascertained from history of COPD or were using long-term inhaled or oral β-agonists, inhaled anti-inflammatory agents, leukotriene receptor antagonists, or inhaled steroids	STEMIs *In-hospital management* Primary percutaneous coronary intervention COPD: 83.1% Non-COPD: 85.4% *P* < 0.001 Overall reperfusion COPD:92.8% Non-COPD: 94.3% *P* < 0.001 *Discharge medicines* Aspirin COPD: 97.8% Non-COPD: 98.7% *P* < 0.001 β-Blocker COPD: 89.4% Non-COPD: 93.1% *P* < 0.001 ACE inhibitor or angiotensin receptor blocker COPD: 78.0% Non-COPD: 78.4% *P* = ‘not statistically significant’ Statin COPD: 92.9% Non-COPD: 94.7% *P* < 0.001 P2Y12 inhibitor COPD: 79.6% Non-COPD: 86.6% *P* < 0.001 NSTEMIs *In-hospital management* Cardiac catheterization COPD: 69.9% Non-COPD:81.2% *P* < 0.001 Percutaneous coronary intervention within 48 hours COPD: 37.2% Non-COPD 48.9% *P* < 0.001 *Discharge medicines* Aspirin COPD: 95.9% Non-COPD: 97.3 *P* < 0.001 β-Blocker COPD: 85.5% Non-COPD: 90.5% *P* < 0.001 ACE inhibitor or angiotensin receptor blocker COPD: 69.6% Non-COPD: 69.6% *P* = ‘not statistically significant’ Statin COPD: 85.9% Non-COPD: 89.5% *P* < 0.001 P2Y12 inhibitor COPD: 65.5% Non-COPD: 71.6% *P* < 0.001
Rothnie *et al.* 2015^18^	All UK patients admitted to hospital in the MINAP registry between 2003 and 2013	300 161 patients with a first MI	STEMI *In-hospital management* Primary PCI OR 0.87 (95% CI 0.83–0.92)^a^ *Discharge medicines* Aspirin OR 0.90 (95% CI 0.85–0.94)^a^ β-Blocker OR 0.26 (95% CI 0.25–0.27)^a^ ACE inhibitor or angiotensin receptor blocker OR 0.89 (95% CI 0.85–0.93)^a^ Statin OR 0.91 (95% CI 0.86–0.95)^a^ P2Y12 inhibitor OR 0.98 (95% CI 0.94–1.03)^a^ NSTEMI *In-hospital management* Angiography in-hospital OR 0.69 (95% CI 0.66–0.71)^a^ *Discharge medicines* Aspirin OR 0.91 (95% CI 0.88–0.94)^a^ β-Blocker OR 0.25 (95% CI 0.24–0.25)^a^ ACE inhibitor or angiotensin receptor blocker OR 0.94 (95% CI 0.91–0.97)^a^ Statin OR 0.93 (95% CI 0.90–0.96)^a^ P2Y12 inhibitor OR 0.97 (95% CI 0.94–1.01)^a^
Salisbury *et al.* 2007^19^	Cohort study in 19 centres in the USA between 2003 and 2004	2481 MI patients in PREMIER study restricted to patients discharged alive after MI	*In-hospital management* Cardiac catheterization COPD: 45.7% Non-COPD: 41.2% *P* = 0.094 Percutaneous coronary intervention COPD: 50.9% Non-COPD: 62.9% *P* < 0.001 *Discharge medicines* Aspirin COPD: 87.8% Non-COPD: 94.5% *P* < 0.001 β-Blocker COPD: 86.2% Non-COPD: 92.6% *P* < 0.001
Stefan *et al.* 2012^17^	Cohort study of patients hospitalized with acute MI at greater Worcester, MA between 1997 and 2007	6290 patients hospitalized with acute MI in greater Worcester, MA medical centres	*In-hospital management* Cardiac catheterization OR 0.56 (95% CI 0.48–0.65)^b^ Percutaneous coronary intervention OR 0.64 (95% CI 0.54–0.77)^b^ *Discharge medicines* β-Blocker OR 0.44 (95% CI 0.35–0.50)^b^ Anticoagulant OR 0.81 (95% CI 0.69–0.95)^b^ Statin OR 0.70 (95% CI 0.60–0.82)^b^ Calcium channel blocker OR 1.31 (95% CI 1.13–1.52)^b^

^a^All ORs compared COPD with non-COPD patients and are adjusted for age, sex, smoking status, and co-morbidities.

^b^ORs compare COPD with non-COPD patients and are adjusted for age, sex, year, cardiovascular disease history, renal failure, length of stay, and type of MI (STEMI or NSTEMI).

## Outcomes after myocardial infarction in people with chronic obstructive pulmonary disease

### All-cause mortality

**Figure 2:**
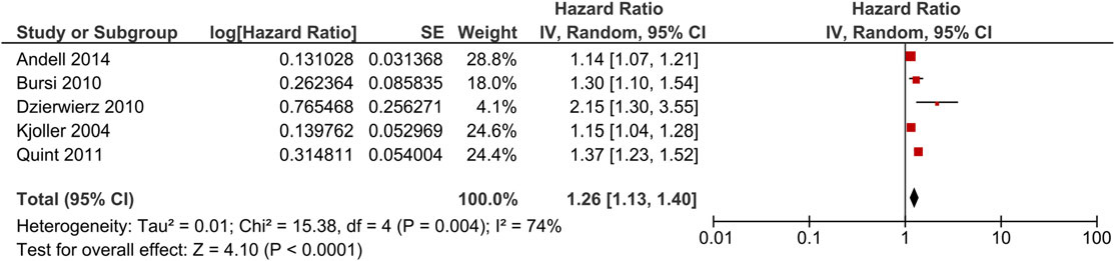
Long-term risk of death following MI comparing chronic obstructive pulmonary disease with non-chronic obstructive pulmonary disease patients. Original image from Rothnie *et al*.^11^

Studies in a variety of settings have demonstrated an increased risk of death during follow-up after AMI for patients with COPD, but whether this applies to in-hospital mortality is less certain, some studies reporting increased mortality^16,17,20,21,29–31^ and others finding no difference^15,32^ compared with patients without COPD. The evidence has now been appraised in a systematic review and meta-analysis,^11^ which concluded that after pooling maximally adjusted estimates from several studies, there is only weak evidence for a difference in in-hospital mortality for patients with COPD (OR 1.13, 95% CI 0.97–1.31) but strong evidence for an increased risk of death during follow-up (HR 1.26, 95% CI 1.13–1.40) (*Figure 2*). However, effects were heterogeneous between studies perhaps because of the international differences in treatment of AMI between patients with and without COPD. If some of the increased risk of death associated with COPD is due to these treatment differences, this is likely to have contributed to the heterogeneous outcomes identified in the systematic review.

**Figure 3:**
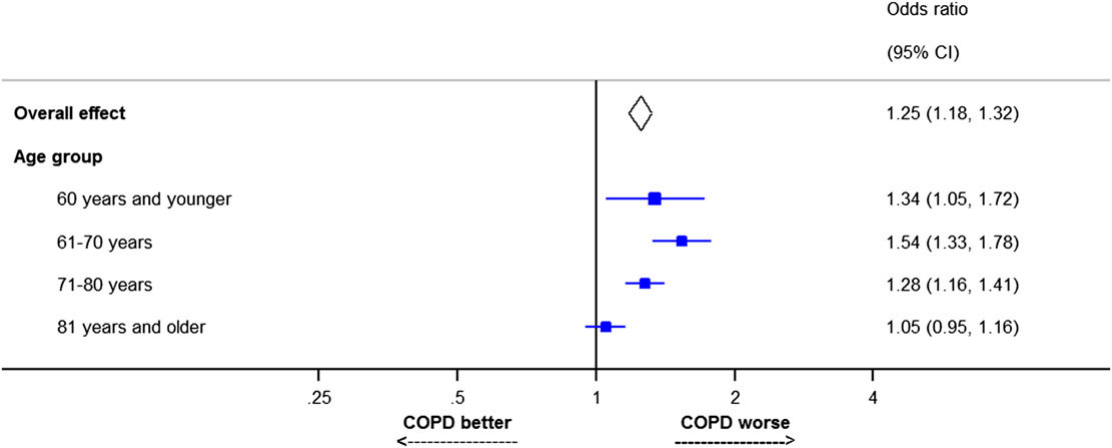
Effect of chronic obstructive pulmonary disease on risk of death 6 months after myocardial infarction split by age group. Adapted from data presented in Rothnie *et al*.^18^

The effect of COPD on risk of death following AMI is modified by mode of presentation, a recent UK study reporting that the adjusted odds of in-hospital and 6-month mortality were higher for NSTEMI [(OR 1.40, 95% CI 1.30–1.52) and (OR 1.63, 95% CI 1.56–1.70)] compared with STEMI [(OR 1.27, 95% CI 1.16–1.39) and (OR 1.43, 95% CI 1.29–1.58)].^18^ Similar findings have been reported in a US study.^20^ The effect of COPD on risk after AMI appears to be greater in younger than in older patients (*Figure 3*), suggesting that the ‘excess’ risk of death, attributable to COPD, is clustered in younger patients.^18^ Dziewierz *et al*.^30^ made a similar observation, reporting that COPD was associated with an increased mortality risk after AMI only in patients aged <75. The increased AMI mortality estimates in studies that have compared patients with and without COPD are likely to be underestimates based on the atypical presentations that characterize these patients, a proportion of whom, no doubt, escape diagnosis altogether. Further contribution to the underestimation of risk in these patients is the absence of data on pre-hospital mortality, all existing studies being confined to patients admitted to hospital.

### Other outcomes

Outcome analyses after AMI in patients with COPD show that the risk of other endpoints, apart from mortality, may also be increased. This applies particularly to heart failure both in the acute phase and after discharge from hospital. Thus, Stefan *et al*.^17^ found that after adjusting for confounders, people with COPD were more likely to experience acute heart failure (OR 1.59, 95% CI 1.37–1.83), compared with patients without COPD, but not atrial fibrillation, cardiogenic shock, or stroke. Similar findings have been reported in unadjusted analyses.^15,20^ Studies of longer-term complications of AMI in patients with COPD confirm that the increased risk of heart failure compared with patients without COPD extends to the chronic phase after discharge from hospital, Andell *et al*. reporting a hazard ratio of 1.35 (95% CI 1.24–1.47) during the first year.^16^ Findings were similar in another study that included patients with heart failure or left ventricular systolic dysfunction, and reported a hazard ratio for admission with heart failure of 1.19 (95% CI 1.05–1.34) among patients with COPD during the first 3 years after AMI.^33^ In the same study, the hazard of sudden death was also higher in patients with COPD (HR 1.26, 95% CI 1.03–1.53), although whether this applies in less selected populations is unclear. Certainly, there is no convincing evidence that patients with COPD are at higher risk of recurrent AMI, stroke, angina, or major bleeds compared with non-COPD patients.^16,19,33^

## Are differences in recognition and management associated with differences in outcomes?

A key question that arises from the tendency of patients with COPD to present atypically and receive under-treatment of AMI is the extent to which this might explain their adverse outcomes, particularly their heightened risk of death and heart failure compared with patients without COPD.

The association of atypical presentation of AMI with adverse outcomes has been previously reported.^34,35^ Patients who present atypically are less likely to receive guideline-recommended reperfusion therapy or invasive management and are less likely to receive β-blockers, statins, or antiplatelet therapy on discharge from hospital.^35^ The tendency of patients with COPD to present with atypical symptoms is, therefore, important because delayed diagnosis of AMI and its under-treatment with reperfusion therapy and secondary prevention drugs has now been shown to explain some of the excess mortality for patients with COPD.^18^ Similar findings have been reported by Andell *et al*.^16^ who found that hazard ratios for mortality in COPD patients fell from 1.32 (95% CI 1.24–1.40) to 1.14 (95% CI 1.07–1.21) following adjustment for in-hospital and discharge treatment. These findings point strongly to delayed diagnosis of AMI and its under-treatment as being important mediators of the adverse outcomes for patients with COPD. They also suggest that differences in treatment between countries may be a plausible reason for heterogeneity in the effects of COPD on risk of death.

**Figure 4:**
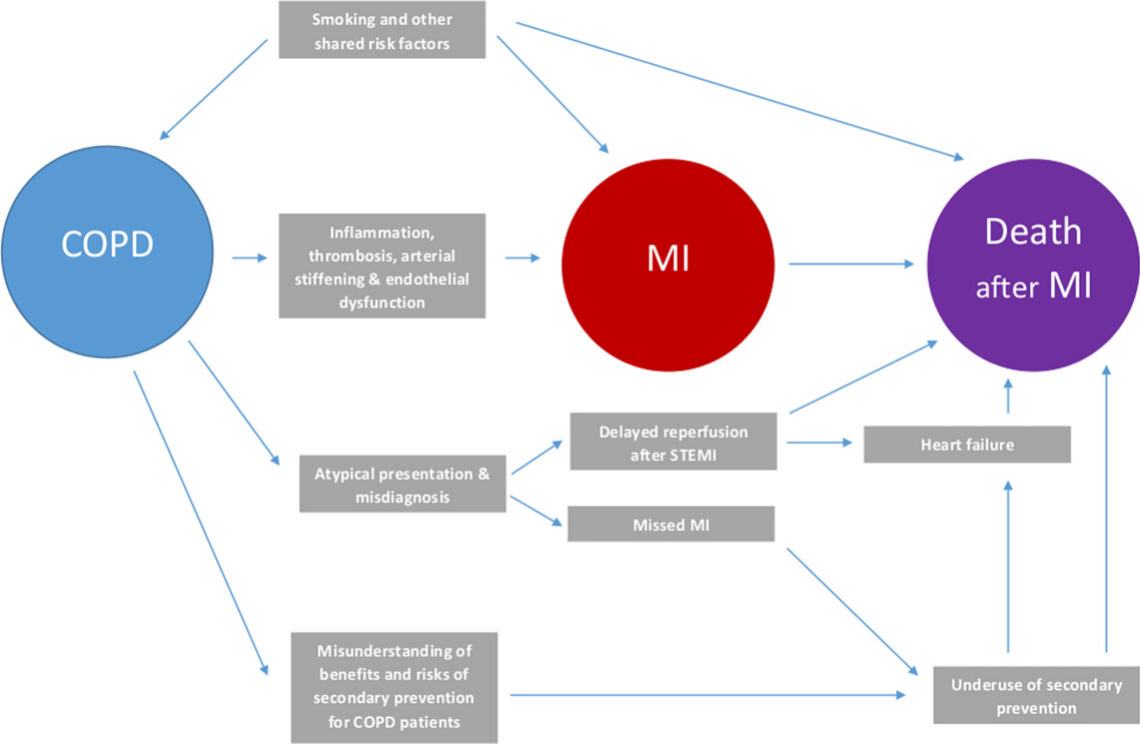
Schematic diagram of the possible mechanisms underlying the relationship between chronic obstructive pulmonary disease and risk of death after acute myocardial infarction.

In considering delayed diagnosis of AMI and its under-treatment as causes of excess mortality, potential direct effects of COPD should not be overlooked (*Figure 4*). Chronic obstructive pulmonary disease severity, defined by degree of airflow obstruction, appears to be a risk factor for AMI,^36^ but lung function data are unavailable in national AMI registries and it is unclear if it is also a risk factor for outcomes after AMI. Exacerbations of COPD, however, and the associated systemic inflammation, are important drivers of mortality,^11,37–39^ but whether ‘frequent exacerbators’ are at heightened risk of death after AMI is uncertain. There is greater certainty about the risks associated with smoking that is often responsible for COPD and is also a major risk factor for death and recurrent coronary events after AMI.^40^ Indeed, quitting smoking after AMI is one of the most effective preventive strategies, but in heavily dependent COPD patients may be hard to achieve. Smoking cessation pharmacotherapy is underused, and although it may be less effective after AMI,^41,42^ the excess mortality in patients with COPD identifies them as a group that needs targeting.

The under-treatment of AMI in patients with COPD is important because it is potentially modifiable, and provides a means of narrowing the mortality gap between patients without COPD. Although under-treatment can be identified across the management spectrum, it is β-blockers that stand out as the drugs that clinicians often avoid for fear of exacerbating airways obstruction, and this despite there being clear evidence that cardio-selective agents are safe for COPD patients with AMI and also effective for secondary prevention. Thus, Quint *et al*.^43^ conducted a propensity matched cohort study in COPD patients with AMI and showed that patients started on a β-blocker during hospital admission had significantly better survival than patients not prescribed a β-blocker (HR 0.50, 95% CI 0.36–0.69). Similar results have been reported for a heart failure population with AMI^33^ in which COPD did not appear to modify the effect of β-blockers on mortality. The continuing reluctance of clinicians to prescribe β-blockers to COPD patients needs addressing because it may drive much of the increased risk of heart failure and death in the months and years following AMI.

Other potential contributors to the risk management paradox^44^ that characterizes AMI patients with COPD include the poor performance of risk algorithms and therapeutic nihilism. Thus, the GRACE score appears to perform less well in patients with COPD, but whether this makes a significant contribution to under-treatment seems unlikely because COPD patients with the same GRACE score as non-COPD patients remain less likely to receive guideline-recommended investigation and treatment.^45^ Potentially more important is therapeutic nihilism whereby COPD patients are seen as too old and frail to make interventional management and secondary prevention worthwhile, even though cardiovascular disease is a leading cause of death in patients with COPD and many of the excess deaths are in younger patients.

## Conclusions

Chronic obstructive pulmonary disease increases the risk of heart failure and death after AMI, particularly in the months after discharge from hospital. Effects are greater in younger patients and those with NSTEMI. Although direct effects of COPD likely contribute to the increased risk, delays in diagnosis and under-treatment are also important. It is the under-treatment of these patients, particularly with β-blockers, that provides the most modifiable target for reducing mortality. Further research is needed to investigate the extent and impact of missed AMI diagnosis in patients with COPD. Researchers should also focus on investigating how AMI risk scores function in COPD and how they should be used to guide treatment.

## Funding

This work was supported by a Medical Research Council Industry Collaboration Agreement (G0902135). Funding to pay the Open Access publication charges for this article was provided by Imperial College London.

**Conflict of interest**: J.K.Q. reports grants from Medical Research Council and GSK during the conduct of the study; grants from Medical Research Council, BLF, Wellcome Trust, and the Chartered Society of Physiotherapy, and personal fees from AZ and GSK, outside the submitted work.
